# Peut-on améliorer la motivation des étudiants en médecine pour un cours fondamental de physiologie en intégrant à l’exposé magistral quelques méthodes pédagogiques actives?

**DOI:** 10.11604/pamj.2017.28.315.10251

**Published:** 2017-12-26

**Authors:** Yassamine Bentata, Catherine Delfosse

**Affiliations:** 1 Département de Néphrologie-Physiologie, Faculté de Médecine et de Pharmacie d’Oujda, Université Mohamed Premier, Oujda, Maroc; 2 Laboratory of Epidemiology, Clinical Research and Public Health, Medical School, University Mohammed The First, Oujda, Morocco; 3 Labset, Université de Liège, Belgique

**Keywords:** Motivation, physiologie, étudiants en médecine, pédagogie active, carte conceptuelle, Motivation, basic education, medical studies, case study, conceptual map

## Abstract

La motivation des étudiants est une condition essentielle à l’apprentissage et la perception qu’a l’étudiant de la valeur qu’il accorde à une activité est l’une des trois composantes majeures de cette motivation. Comment amener les étudiants à percevoir l’utilité et l’intérêt de leurs cours universitaires tout en suscitant leur motivation ? L’objectif de l’étude est de déterminer la perception de la valeur attribuée par les étudiants au cours fondamental de physiologie et d’évaluer l’impact de l’intégration de quelques méthodes d’apprentissage actif aux exposés magistraux sur la motivation des étudiants du premier cycle des études médicales dans une jeune faculté. Cette étude prospective, a concerné les étudiants de deuxième année de médecine (PCEM2). Nous avons procédé initialement à une appréciation de la perception de la motivation des étudiants pour les cours universitaires via un premier questionnaire, après nous avons intégré deux activités pédagogiques: l’étude de cas et la réalisation de carte conceptuelle aux exposés magistraux du module de physiologie et à la fin nous avons évalué via un deuxième questionnaire l’impact de ces deux activités sur la motivation des étudiants. 131 et 109 étudiants ont rempli et rendu respectivement le 1^er^ et le 2^ème^ questionnaire parmi les 249 étudiants inscrits en PCEM2. La motivation de nos étudiants à l’égard de leurs cours universitaires était globalement très favorable, même si la motivation pour le cours de physiologie (70,8%) était légèrement moins importante que pour l’ensemble des cours (80%). Nos étudiants avaient apprécié les deux activités proposées et seulement 13% (pour l’étude de cas) et 16,8% (pour la carte) ne se montraient pas satisfaits. 40,9% des étudiants avaient réalisé une carte conceptuelle et la qualité de ces productions, jugée sur l’identification des concepts et des liens inter concepts, était globalement satisfaisante pour une première expérience. Influencée par de multiples facteurs internes et externes, la motivation des étudiants reste une grande problématique en milieu universitaire. Dans ce contexte, une planification rigoureuse d’activités pédagogiques diversifiées et actives est l’une des principales portes offertes à l’enseignant pouvant susciter cette motivation.

## Abstract (en)

Students’ motivation is a critical component of learning and students’ perception of activity value is one of the three major components of their motivation. How can we make students perceive the usefulness and the interest of their university courses while increasing their motivation? The aim of our study was to determine students’ perception of basic physiology education value and to assess the impact of lecture integration into some active learning methods on the motivation of the students of the first cycle of Medicine in a junior faculty. We conducted a prospective study, involving the students in their second year of medical studies. At first, we assessed students’ motivation for university courses through a first questionnaire, after we integrated two educational activities: the case study and the realization of a conceptual map for the lectures of the physiology module and then we evaluated, through a second questionnaire, the impact of these two activities on students’ motivation. Out of 249 students in their second year of medical studies 131 and 109 students have completed and returned the 1st and 2nd questionnaire respectively. Overall students’ motivation for their university courses was very favorable, even if the motivation for physiology course (70.8%) was slightly lower than for all the courses (80%). Our students enjoyed the two proposed activities and only 13% (for the case study) and 16.8% (for the map) were not satisfied. 40.9% of students completed a conceptual map whose quality judged on the identification of concepts and of the links between concepts was globally satisfactory for a first experience. Students’ motivation is influenced by multiple internal and external factors and is a big problem in the university environment. In this context, a rigorous planning of diversified and active educational activities is one of the main gateways for teacher to encourage motivation.

## Introduction

**Figure 1:**
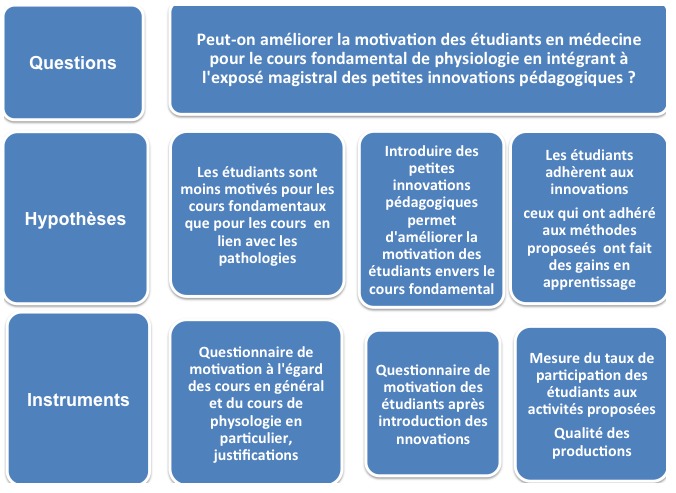
Question, hypothèses et instruments émis pour la méthodologie de l’étude

La motivation des étudiants est une condition essentielle à l’apprentissage. Elle a été l’objet de nombreux travaux qui ont consisté en l’identification de ses différents composants, l’analyse de son impact réel sur le processus d’apprentissage et l’élaboration des méthodologies pédagogiques pour la susciter et l’entretenir et ceci à tous les niveaux d’enseignement du primaire à l’universitaire. La motivation reste un paramètre complexe à étudier car elle est largement influencée par de multiples facteurs externes et internes. La perception qu’à l’étudiant de lui même et du contexte dans lequel se déroule son apprentissage est l’un des composants les plus importants à considérer dans ce contexte. Viau dans son modèle de la dynamique motivationnelle considère trois éléments: la perception qu’à l’étudiant de la valeur qu’il accorde à une activité, la perception qu’il a de sa compétence à réussir cette activité et sa perception du degré de contrôle qu’il exerce sur le déroulement et les conséquences de celle-ci [1-6]. Ces déterminants motivationnels influencent trois comportements d’apprentissage: l'engagement cognitif qui correspond au degré d'effort mental que l’étudiant déploie lors de l'exécution d'une activité d'apprentissage, la persévérance qui se traduit par le temps qu’il lui consacre et enfin la performance qui désigne les résultats obtenus [7]. Quel que soit l’élément déterminant en cause, la démotivation a des conséquences importantes: démotivés, les étudiants ne s’engageront pas et ne persévéreront pas dans les cours, la procrastination et/ou les notes faibles qui en résultent conduiront un bon nombre d’entre eux à échouer et/ou à abandonner leur études. Nous nous sommes intéressés au premier élément de cette dynamique motivationnelle qu’est la perception de la valeur d’une activité, autrement dit au jugement que l’étudiant porte sur l’utilité et l’intérêt d’une activité en vue d’atteindre les buts qu’il poursuit. Pour comprendre notre intérêt pour ce déterminant, il est important de présenter la problématique de l’enseignement des études médicales dans notre contexte. L’enseignement des études médicales universitaires a pour principale finalité l’acquisition des compétences requises pour l’exercice de la médecine générale. Cet enseignement s’organise en deux cycles: un premier cycle qui comprend des modules de sciences fondamentales et un deuxième cycle qui comprend des modules de sémiologie et de pathologie médico-chirurgicale. L’étudiant reçoit ainsi deux enseignements distincts en bloc sans véritables liens entre théorie et pratique et perçoit peut-être l’enseignement du premier cycle comme un enseignement peu utile et déconnecté par rapport à l’image qu’il se fait de sa future profession de médecin. Cette impression est renforcée par le fait que l’enseignement des sciences fondamentales est un enseignement qui se déroule dans sa presque totalité sous forme de cours magistraux classiques au sein des grands amphithéâtres de la faculté et destiné à des centaines d’étudiants alors que l’enseignement du deuxième cycle s’apprête à des activités pédagogiques variées et actives, s’accompagne d’un encadrement pratique parallèle en milieu hospitalier et est destiné à des petits groupes d’étudiants. Cet environnement contribue ainsi à susciter la motivation des étudiants et à créer un climat attractif et propice au développement des compétences disciplinaires. L’objectif de ce travail est de déterminer la perception de la valeur attribuée par les étudiants au cours de physiologie et d’évaluer l’impact de l’intégration de méthodes d’apprentissage actif aux exposés magistraux sur la motivation des étudiants du premier cycle des études médicales dans une jeune faculté du Royaume du Maroc. Enfin, nous tenterons de recueillir des éléments en lien avec d’éventuels gains en apprentissage de la part des étudiants. Les hypothèses et les instruments de ce travail sont présentés dans la Figure 1.

## Méthodes

Il s’agit d’une étude prospective, étalée sur trois mois (Février 2013- Avril 2013) et menée au niveau de la Faculté de Médecine et de Pharmacie d’Oujda, située à l’est du Maroc. Il s’agit d’une jeune faculté qui a ouvert ses portes en 2008 et l’enseignement y est dispensé principalement dans une approche cours. Cette étude a concerné les 249 étudiants de la deuxième année du premier cycle des études médicales (PCEM2). Le module de physiologie est dispensé principalement sous forme de cours magistraux. Dans ce contexte, l’enseignement reste principalement transmissif et parce que les étudiants sont très passifs, qu’ils ne perçoivent pas les liens entre données théoriques et pathologies et par voie de conséquence se démotivent et ne s’investissent pas ou peu dans les cours.

Notre intervention a consisté dans un premier temps à mettre sur pieds deux activités au sein des exposés magistraux concernant les deux modules de physiologie: physiologie des reins et physiologie du système endocrinien. La première innovation visait à proposer des situations authentiques sous forme d’un cas clinique ainsi que d’élaborer d’une carte conceptuelle dont le thème abordé était « l’adaptation de l’organisme à une situation de stress ». Cette forme d’appropriation devrait aider les étudiants à percevoir l’utilité des données théoriques dans leur pratique. Les étudiants étaient libres de réaliser cette dernière activité individuellement ou en groupe (maximum 5 par groupe). Les cartes élaborées étaient jugées correctes et complètes quand elles reproduisaient les concepts et les liens inter concepts exigés par le thème. Une séance était dédiée à la projection d’une dizaine de cartes réalisées par les étudiants et comparées à celle de « référence » élaborée par l’enseignant. Et pour répondre à notre question « Peut-on améliorer la motivation des étudiants en médecine pour un cours fondamental de physiologie en intégrant à l'exposé magistral des petites innovations pédagogiques? », nous avons émis et tenté de vérifier les trois hypothèses suivantes: 1) Les étudiants sont moins motivés pour les cours fondamentaux que pour les cours en lien avec les pathologies; 2) Intégrer des innovations pédagogiques comme l’étude de cas et la réalisation de carte conceptuelle est susceptible d’augmenter la motivation des étudiants en leur permettant de comprendre les liens entre données théoriques et cliniques; 3) L’amélioration du cours magistral par l’introduction de méthodes actives est susceptible de provoquer des gains en apprentissage en suscitant d’abord la motivation des étudiants et en s’investissant plus secondairement.

Ensuite, nous avons tenté de vérifier les trois hypothèses sus citées et reprises dans la Figure 1 et nous avons collecté les données suivantes: Hypothèse 1: afin d’objectiver notre intuition, nous avons considéré les taux de présence des étudiants lors des cours universitaires en général et lors de notre cours de physiologie en particulier et nous les avons également interrogé via un premier questionnaire de motivation. Ce questionnaire a été élaboré en nous basant sur des questionnaires existants et que nous avons adaptés à notre contexte [8-10]. Ce questionnaire papier comprenait 26 items. Hypothèse 2: afin de vérifier si l’intégration des innovations pédagogiques comme l’étude de cas et la réalisation de carte conceptuelle est susceptible d’augmenter la motivation des étudiants, nous avons réinterrogé les étudiants via un deuxième questionnaire analogue au premier et portant sur leur motivation à suivre le cours de physiologie dans sa forme améliorée. Ce questionnaire papier comprenait 14 items liés à la motivation, à la satisfaction et à l’intérêt pour l’apprentissage des innovations apportées. Hypothèse 3: pour confirmer ou infirmer si les étudiants ont réellement adhéré aux petites innovations et si l’amélioration du cours magistral par l’introduction de ces innovations est susceptible de provoquer des gains en apprentissage, nous nous sommes donc, comme présenté dans le schéma de la Figure 1, intéressés au taux de participation des étudiants à l’élaboration des cartes conceptuelles et à l’appréciation de la qualité de celles-ci. Nous avons recueilli l’ensemble des cartes réalisées par les étudiants et nous avons comptabilisé le nombre de concepts et de liens inter concepts reproduits sur ces cartes en les comparant à une carte « de référence » produite par l’enseignant.

## Résultats

**Figure 2:**
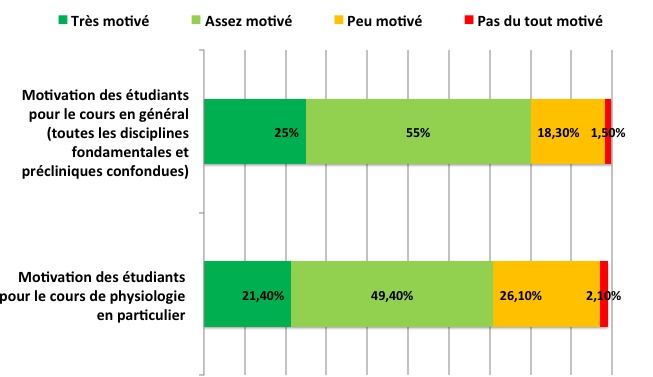
Motivation des étudiants pour les cours universitaires en général et pour le cours de physiologie en particulier avant intervention (n = 131 étudiants)

**Figure 3:**
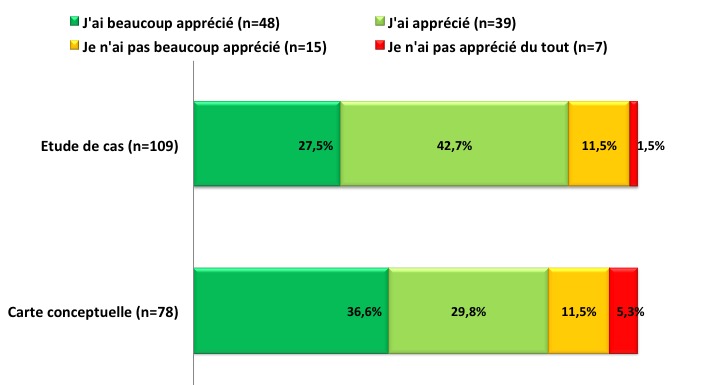
Appréciation de l’étudiant pour l’étude de cas et de la réalisation de la carte conceptuelle

**Figure 4:**
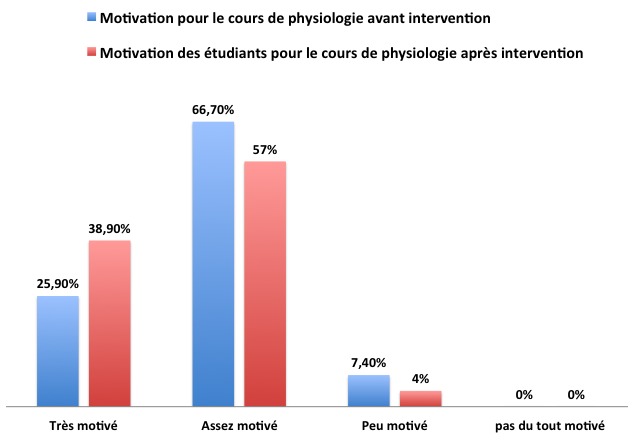
Motivation des étudiants pour le cours de physiologie avant et après intervention (n = 54)

Sont inscrits en deuxième année des études médicales (PCEM2) à la Faculté de Médecine d’Oujda au Maroc, 249 étudiants avec une part plus importante (60,6%) des étudiants du genre féminin. 52,6% (n = 131) des étudiants étaient présents lors de la distribution du premier questionnaire et 43,7% pour le second. Interrogés sur leur motivation via le premier questionnaire à suivre les cours universitaires en général et le cours de physiologie en particulier dans sa version traditionnelle avant intervention, les étudiants fournissent les réponses rapportées dans la Figure 2. Les résultats de l’appréciation des étudiants pour l’étude de cas et la carte conceptuelle sont rapportés dans la Figure 3. 54 étudiants avaient répondu à la fois au premier et au deuxième questionnaire. Parmi ces 54 étudiants, 42 avaient réalisé une carte conceptuelle. Nous avons ainsi comparé la motivation chez ce groupe d’étudiants avant et après introduction des deux innovations pédagogiques. Les résultats sont rapportés dans la Figure 4. Les raisons invoquées par les étudiants concernant leur intérêt pour les deux activités pédagogiques proposées sont rapportées dans le Tableau 1 pour l’étude de cas) et le Tableau 2 pour la carte conceptuelle. Les pistes d’amélioration des cours proposées spontanément par les étudiants sont rapportées dans le Tableau 3. Ces suggestions sont classées à posteriori en lien avec les trois éléments du cours (objectifs, méthodes et évaluation).

**Tableau 1:** Arguments des étudiants justifiant leur appréciation de l’étude de cas

Etude de cas (N=109)	N (%)
**Etude de cas appréciée (n = 92) pour**	
- Illustre bien les liens théorie-pratique	81 (79,4)
- Initie à la compréhension de la pathologie	31 (27,6)
**Etude de cas non appréciée (n =17) pour**	
- La méconnaissance de certains prérequis indispensables	14 (29,7)
- Du temps qui lui était consacré	17 (36,1)
- Des exiges en terme de raisonnement structuré et de réflexion auquel les étudiants se jugent peu préparés	16 (34,1)

**Tableau 2:** Arguments des étudiants ayant réalisé la carte conceptuelle justifiant leur appréciation (n=78)

Etudiants identifiés ayant réalisé la carte conceptuelle et ayant répondu au 2^ème^ questionnaire (N= 78)	N (%)
**Carte conceptuelle appréciée parce qu’elle: (94 citations de 74 étudiants)**	
Permet une synthèse et une intégration générale de plusieurs éléments	69 (88,4)
Une nouvelle méthode auquel les étudiants ne sont pas habitués	14 (17,9)
Favorise le travail avec les condisciples	11 (14,1)
**Carte conceptuelle non appréciée parce qu’elle est: (63 citations de 39 étudiants)**	
Difficile à réaliser	31 (49,2)
Nécessite un temps important pour sa réalisation	19 (30,1)
Les étudiants étaient déconcentrés en raison de l’approche des examens de fin d’année	13 (20,6)

**Tableau 3:** propositions des étudiants pour améliorer le cours

N= 131 étudiants ayant proposés 256 pistes d’amélioration	N (%)
**Les Objectifs du cours: devraient être**	
Limités et précis	31 (12,1)
Facilement atteignables	17 (6,6)
**Dans les méthodes d’enseignement: Il faudrait**	
Plus d’interactivité durant l’exposé magistral	**45 (17,5)**
Davantage de travaux dirigés et de travaux pratiques	20 (7,8)
Des exercices à réaliser	22 (8,6)
Des illustrations (vidéo, schéma, tableaux, carte conceptuelle)	**67 (26,1)**
Davantage de liens théorie-pratique	**81 (31,5)**
**En ce qui concerne l’’Evaluation**	
Instaurer une évaluation formative	15 (5,8)
Préciser clairement les modalités de l’évaluation sanctionnante	39 (15,2)

102 étudiants ont participé à la réalisation de 34 cartes conceptuelles. Sept cartes conceptuelles ont été réalisées individuellement et 27 par des groupes constitués de 3 à 5 étudiants. Ces cartes ont été réalisées sur papier. Parmi les 109 étudiants ayant répondu au deuxième questionnaire, 82 (75,9%) avaient mentionné leur intention de réutiliser la technique de la carte conceptuelle dans les autres cours.Vingt-trois cartes conceptuelles parmi les 34 réalisées reproduisaient l’ensemble des concepts exigés par le thème mais seulement 14 reproduisaient les liens inter concepts attendus. En raison de la non adhésion de certains étudiants et le manque de perfection des cartes conceptuelles produites, nous avons analysé deux autres éléments non liés directement aux cours mais pouvant influencer la motivation des étudiants à adhérer aux innovations pédagogique à savoir la maitrise de la langue française et de la technique de prise des notes par nos étudiants. Parmi les 131 étudiants ayant répondu au premier questionnaire, 28,5% reconnaissaient avoir des difficultés à maîtriser la langue française et 54,1% déclaraient avoir des difficultés à prendre des notes. Ces éléments peuvent-ils jouer un rôle dans l’adhésion des étudiants aux innovations proposées? Pour tenter de répondre à cette question nous avons comparé la difficulté de maitrise de langue française et de la technique de prise de note chez étudiants ayant réalisé la carte vs étudiants n’ayant pas réalisé de carte parmi les 54 étudiants identifiés. Nos résultats sont rapportés dans le Tableau 4.

**Tableau 4:** difficulté de maitrise de la langue française et de la technique de prise de notes (n=54)

	Etudiants ayant réalisé la carte conceptuelle (N=42)	Etudiants n’ayant pas réalisé la carte conceptuelle (N=12)	p
Difficulté de maitrise de la langue française, n (%)	5 (11,9)	4 (33,3)	0,09
Difficulté de la technique de prise de notes, n (%)	22 (52,4)	9 (75)	0,14

## Discussion

**Nos résultats en réponse à l’hypothèse 1 montrent que** La motivation de nos étudiants à l’égard de leurs cours universitaires est globalement très favorable, même si la motivation pour le cours de physiologie est légèrement moins importante que pour les cours en général. Le taux de présence des étudiants aux cours magistraux, moins encourageant en 2^ème^ cycle qu’en 1^er^ cycle des études médicales, reflète: *En première ligne:*l’existence d’une réelle motivation des étudiants du 1^er^ cycle qui peut être expliqué par l’enthousiasme, la fierté et la curiosité des étudiants à découvrir les études universitaires médicales tant convoitées. *En deuxième ligne:*une perte de la motivation des étudiants. Cette décroissance de motivation au fil des années d’études universitaires a été également observée par Viau [11]. Viau explique ce phénomène par « l’essoufflement » des étudiants, les changements personnels d’ordre financier ou familial et/ou des facteurs liés directement au cours tels que les activités pédagogiques, les pratiques évaluatives, la relation avec l’enseignant et le climat de la classe. En contexte médical en particulier, ce phénomène pourrait être expliqué par le fait que les étudiants deviennent démotivés pour les cours magistraux au profit du nouvel intérêt qu’ils portent pour l’apprentissage pratique en milieu hospitalier.

Peut-on conclure que nos étudiants sont réellement motivés à travers nos résultats? D’une part, il est important de rappeler que seuls les étudiants présents à la séance du cours avaient répondu au questionnaire et avaient par conséquent exprimé leur perception de la motivation. Nous ignorons complétement la perception de la motivation des étudiants absents le jour de distribution du questionnaire et qui étaient au nombre de 118 soit 47,3% des étudiants inscrits. D’autre part, notre étude a été menée en fin d’année et à l’approche de la période des examens, ce qui explique le taux élevé d’absence aux cours magistraux observé en cette période, d’autant plus que la présence à ces cours n’est pas obligatoire. S’ajoute à ces éléments la grande désirabilité sociale liée à la question sur la perception de la motivation qui ne laisse pas un libre choix aux étudiants et au fait qu’il s’agissait de l’enseignante de la matière qui évalue elle même la matière qu’elle dispense auprès de ses étudiants via un questionnaire non anonyme. Les recherches sur la motivation en contexte d’apprentissage se sont développées essentiellement au Canada et se sont initialement intéressées au milieu scolaire [1,8]. Progressivement, l’épineux constat du taux d’échecs au niveau des études supérieures a débouché sur des études mettant en évidence l’importance des facteurs motivationnels. La problématique de la motivation des étudiants ne se présente pas de la même façon à l’université qu’au secondaire. Alors qu’un grand nombre d’élèves souffre de démotivation dès leur arrivée au secondaire, les étudiants universitaires, quant à eux, rentrent généralement à l’université avec une forte motivation et un grand enthousiasme mais qui décroissent de façon rapide et importante au fil des années d’étude. Très peu d’études sont disponibles concernant la motivation des étudiants universitaires en milieu médical et encore moins concernant les cours fondamentaux du premier cycle. C’est ainsi que nous nous sommes intéressés à la motivation des étudiants du premier cycle des études médicales pour le cours fondamental de physiologie partant d’une intuition que ce cours était perçu comme peu utile dans la pratique de profession et par voie de conséquence peu motivant pour les étudiants. Nos résultats ont révélé que nos étudiants sont globalement motivés à suivre le cours fondamental, mais ces résultats sont à prendre avec prudence en raison des arguments évoqués ci dessus. Pourquoi les étudiants universitaires sont démotivés? En analysant les principaux facteurs traités dans la littérature, on retrouve: les activités pédagogiques, les pratiques évaluatives, la relation avec l’enseignant et le climat de la classe [11]. Et nous pensons que dans notre contexte aussi, la démotivation des étudiants semble être liée aux activités pédagogiques proposées par le professeur, que les étudiants jugent peut-être inadaptées ou peu adaptées.

**Nos résultats en réponse à l’hypothèse 2 montrent que** Nos étudiants ont globalement apprécié les deux activités proposées: l’étude de cas et la réalisation de la carte conceptuelle. Et pour répondre aux attentes des étudiants et comme nous avons pressenti avant de débuter l’étude leurs difficultés à établir des liens théorie-pratique, nous avons décidé d’apporter des petites innovations pédagogiques axées sur la mise en valeur des liens théorie-pratique tout en gardant l’exposé magistral. Nous pensons que l’exposé magistral garde toujours sa place dans l’enseignement universitaire et particulièrement dans notre contexte où le nombre d’étudiants est important et le nombre d’enseignants qualifiés est insuffisant et nous y avons intégré deux petites innovations pédagogiques, l’étude de cas et la réalisation d’une carte conceptuelle. Nous avons choisi l’étude de cas car cette activité permet de contextualiser le savoir théorique dans différentes situations authentiques et la carte conceptuelle quant à elle permet d’intégrer l’ensemble des concepts théoriques et pratiques et d’identifier les liens inter concepts. Viau à travers une étude menée en milieu universitaire observe que l’approche par projet, les études de cas et l’approche par problème sont les activités que les étudiants perçoivent comme étant les plus utiles [10]. Ceci est lié au fait que ces trois activités proposent aux étudiants des situations authentiques comme celles susceptibles de rencontrer dans la future vie professionnelle. Les étudiants ont peut-être davantage apprécié la carte conceptuelle que l’étude de cas parce que d’une part l’étude de cas exige de grandes connaissances multidisciplinaires et que le raisonnement clinique approfondi qui en découle ne peut être acquit qu’après un entrainement intensif au raisonnement clinique par des méthodes pédagogiques actives telle que l’apprentissage au raisonnement clinique. D’autre part, la carte conceptuelle en tant qu’activité pédagogique active offre à l’étudiant la possibilité de prendre en main son apprentissage du fait qu’elle soit signifiante à ses yeux, représente un défi pour lui, lui permet d’interagir et de collaborer avec les autres, comporte des consignes claires, le responsabilise en lui permettant de faire un choix et se déroule dans une période de temps suffisante. L’ensemble de ces caractéristiques sont susceptibles de susciter et de maintenir la motivation des étudiants [12-15]. Tous ces éléments contribuent ainsi à animer la dynamique motivationnelle de l’étudiant lorsqu’il accomplit cette activité.

**Nos résultats en réponse à l’hypothèse 3 montrent que** Le taux d’adhésion de nos étudiants à la réalisation de la carte ainsi que la qualité de leurs productions jugée sur l’identification des concepts et des liens inter concepts sont globalement satisfaisants. Les étudiants ayant réalisé la carte se sont probablement rendu compte que cette activité leur permettrait également de synthétiser le cours et que le document produit leur servirait de support pour l’apprentissage durant l’année et de support de révision pour les examens. Ceci expliquerait entre autres pourquoi nos étudiants souhaiteraient réutiliser la carte dans d’autres modules d’enseignement.

## Conclusion

L’enseignement est une tache complexe faisant intervenir deux principaux acteurs: l’enseignant et l’étudiant. Si l’enseignant a l’obligation de dispenser un enseignement de qualité, l’étudiant quant à lui à l’obligation d’apprendre et de réussir. Atteindre ces objectifs exige que l’enseignant soit dévoué et soucieux de ses étudiants et que l’étudiant soit motivé et engagé, la motivation paraît ainsi au centre de ce processus d’apprentissage. Comment ainsi susciter et maintenir la motivation des étudiants dans un monde universitaire actuel en profonde mutation culturelle, sociale, politique et technologique et aux exigences très élevées? Dans ce contexte, une planification rigoureuse d’activités pédagogiques actives et diversifiées reste l’une des principales portes offertes aux enseignants pour motiver leurs étudiants.

### Etat des connaissances actuelles sur le sujet

- La motivation est un paramètre indispensable à l’apprentissage;
- La perception de l’étudiant pour l’utilité et l’intérêt de l’activité pédagogique est un déterminant majeur de la dynamique motivationnelle;
- La pédagogie active favorise le développement des compétences.

### Contribution de notre étude à la connaissance

- Les liens théories - pratiques suscitent la motivation des étudiants;
- Les petites activités pédagogiques actives permettent d’améliorer la motivation des étudiants;
- Les cartes conceptuelles sont un modèle pédagogique intéressant en médecine.

## Conflits d’intérêts

Les auteurs ne déclarent aucun conflit d’intérêts.

## References

[cit0001] Viau R (1994). La motivation en contexte scolaire.

[cit0002] Wigefield A, et Eccles JS (1992). The development of achievement task values: a theoretical analysis. Develop mental review..

[cit0003] Bandura A (1993). Perceived self-efficacity in cognitive development and functionning. Educational Psychologist..

[cit0004] Bouffard-Bouchard T, Parent S, Larivée S (1991). Influence of self-efficacity on self-regulation and performance among junior and senior high school age students. International Journal of Behavioral Development..

[cit0005] Pajares F (1996). Self-efficacity beliefs in academic setting. Review of Educationnal Research..

[cit0006] Skinner EA (1995). Perceived control, motivation and coping.

[cit0007] Salomon G (1983). The differencial investment of mental effort in learning from different sources. Educational Psychologist..

[cit0008] Vallerand RJ, Blais MR, Briere NM, Pelletier LG (1989). Construction and validation of the motivation towardeducationscale. Canadian Journal of Behavioural Science Revue..

[cit0009] Viau R (2001). La motivation des étudiants à l’université: mieux comprendre pour mieux agir.

[cit0010] Darien M (2012). Profil et motivation des étudiants inscrits à un diplôme universitaire en 2011. Annales françaises d’anesthésie et de réanimation..

[cit0011] Rolland Viau, Jacques Joly, Denis Bédard (2004). La motivation des étudiants en formation des maitres à l’égard d’activités pédagogiques innovatrices. Revue des sciences de l’éducation..

[cit0012] Stipek D (1998). Motivation to learn: from theory to pratice.

[cit0013] Paris SG, et Turner JC, Pintrich PR, Brown DR, et Weinstein CE Situated motivation. Student motivation, cognition, and learning.

[cit0014] Mc Combs BL, Pope JE (1994). Motivating hard to reachstudents.

[cit0015] Brophy J (1998). Motivating students to learn.

